# Outbreak of Respiratory Disease Due to Bovine Respiratory Syncytial Virus with Concomitant Infections by *Histophilus somni* and *Pasteurella multocida* in Adult Dairy Cows and Calves from Southern Brazil

**DOI:** 10.3390/ani15203015

**Published:** 2025-10-17

**Authors:** João Henrique Perotta, Isabela Vaz Silva, Maria Constanza Rodriguez, Mara Eliza Gasino Joineau, Marcel Kruchelski Tschá, Renato Silva de Sousa, Alais Maria Dall Agnol, Flávia Helena Pereira Silva, Sébastien Buczinski, Selwyn Arlington Headley, Ivan Roque de Barros Filho

**Affiliations:** 1 Department of Veterinary Medicine, Federal University of Paraná, Rua dos Funcionários, 1540, Curitiba 80035-050, Paraná, Brazil; perotta@ufpr.br (J.H.P.); aisabelavaz@gmail.com (I.V.S.); renatosousa@ufpr.br (R.S.d.S.); 2 Marcos Enrietti Diagnostic Center, Paraná Agricultural Defense Agency, Rua Jaime Balão, 575, Curitiba 80040-340, Paraná, Brazil; mariaconstanza@adapar.pr.gov.br (M.C.R.); mgasino@adapar.pr.gov.br (M.E.G.J.); 3 Instituto de Biologia Molecular do Paraná (IBPM), Rua Professor Algacyr Munhoz Mader, 3775, Curitiba 81350-010, Paraná, Brazil; tschamarcel@gmail.com; 4 Laboratory of Animal Virology, Department of Veterinary Preventive Medicine, Universidade Estadual de Londrina, Rodovia Celso Garcia Cid, PR 445 Km 380, Londrina 86057-970, Paraná, Brazil; alaisagnol@uel.br; 5 Laboratory of Animal Pathology, Department of Veterinary Preventive Medicine, Universidade Estadual de Londrina, Rodovia Celso Garcia Cid, PR 445 Km 380, Londrina 86057-970, Paraná, Brazil; flaviahelena.pereira@uel.br (F.H.P.S.); selwyn.headley@uel.br (S.A.H.); 6 Département des Sciences Cliniques, Faculté de Médecine Vétérinaire, Université de Montréal, St-Hyacinthe, QC J2S 2M2, Canada; s.buczinski@umontreal.ca

**Keywords:** bovine respiratory disease, diagnostic immunohistochemistry, interstitial pneumonia, multiplex qPCR, molecular identification, non-vaccinated dairy cattle

## Abstract

**Simple Summary:**

There are several reports of clinical outbreaks of Bovine Respiratory Syncytial Virus (BRSV) associated disease in calves but limited documented information in adult cattle. This report describes an outbreak of respiratory disease in adult dairy cows from Southern Brazil due to simultaneous infections by BRSV, *Histophilus somni*, and *Pasteurella multocida*. Nasal discharge, cough, and dyspnea were present in all categories of animals; two cows had clinical manifestations suggestive of subcutaneous emphysema. Paired oral and nasal swabs were used in quantitative multiplex PCR assays designed to identify pathogens of bovine respiratory disease (BRD). Organs from one cow that died was used in histopathological and immunohistochemical (IHC) analyses. Histopathology revealed interstitial pneumonia with intralesional tissues antigens of BRSV by IHC. The multiplex PCR assay detected elevated values of BRSV in most animals, with varying loads of *H. somni* and *P. multocida*. This PCR assay can be used for the fast and accurate detection of pathogens associated with BRD and represents the first utilization of multiplex PCR assay for the diagnosis of BRD in Brazil.

**Abstract:**

Although bovine respiratory syncytial virus (BRSV) is a key contributor to bovine respiratory disease (BRD) worldwide, there are few detailed reports of BRSV-related outbreaks in Brazil. This study describes the clinical, pathological, immunohistochemical (IHC), and molecular findings from a BRD outbreak in adult dairy cows from Southern Brazil. The affected cattle had dyspnea, nasal discharge, and coughing. One cow died, and samples were collected for diagnosis. Histopathology revealed interstitial pneumonia with multinucleated giant cells. IHC identified BRSV antigens in pulmonary tissue. A multiplex real-time PCR identified BRSV, *Histophilus somni*, and *Pasteurella multocida* in nasal and oral swabs, while only BRSV and *H. somni* were detected in the tissues of the cow that died. All animals had co-infections involving BRSV. The average cycle threshold (*Cq*) values for BRSV were 27.43 (nasal) and 32.68 (oral), with significant differences (*p* = 0.016), indicating higher nasal shedding. This qPCR assay was effective for detecting BRD pathogens, the quantification of viral and bacterial loads in animals with BRD and can be used for the rapid detection of respiratory pathogens. The elevated BRSV detection in oral samples suggests that this route may be an alternative for the collection of samples in cattle with profuse nasal discharge.

## 1. Introduction

Bovine respiratory disease (BRD) is a multietiological disease entity that is caused by several pathogens in association with abrupt changes in environmental, nutritional, climatic, and management activities [1,2,3]. The pathogens most frequently associated with BRD include bovine respiratory syncytial virus (BRSV), bovine alphaherpesvirus 1 (BoAHV1), bovine parainfluenza virus 3 (BPIV3), bovine viral diarrhea virus (BVDV), bovine coronavirus (BCoV), *Histophilus somni*, *Pasteurella multocida*, *Mannheimia haemolytica*, and *Mycoplasma bovis* [2,3,4]. Recent studies have shown that ovine gammaherpesvirus 2 (OvGHV2) may also play a key role in the pathogenesis of BRD [5].

BRSV (*Orthopneumovirus bovis*), is a member of the *Orthopneumovirus* genus, family *Pneumoviridae* [6]. BRSV has been associated with the development of BRD since the 1970’s [7], and is the cause of more than 60% of respiratory outbreaks in dairy herds worldwide [8]. BRSV was initially detected in Brazil from pulmonary samples obtained from cattle submitted for slaughter in 1993 [9], and then subsequently isolated from calves with clinical manifestations of respiratory disease [10]. Serological positivity against BRSV is now frequently diagnosed in Brazil, with at least 80% seroprevalence in dairy cows [11,12], and 84% seropositivity in dairy herds [12].

The risk of infection by BRSV is influenced by temperature fluctuation, stress-related events [13], dusty environments, and crowding [14,15], and outbreaks are more prevalent during cold seasons [11,16]. Calves less than one year of age may be more susceptible to infection due to the lack of immunity, while the occurrence of infection in cows is difficult to evaluate due to the elevated seroprevalence in this category of animals [8,15]. However, a study in Brazil revealed that adult cattle may have a great risk of being infected [11].

The incubation period for BRSV varies between 2 and 5 days [8,17]. Clinical signs associated with BRSV can be observed in animals of all ages and may vary from minimal to severe, including tachypnea, fever, and serous to mucopurulent nasal discharge [8,14]. Animals that are severely affected may demonstrate dyspnea and subcutaneous emphysematous bullae [8]. Cows affected frequently have pulmonary manifestations of disease, with typical gross manifestations of interstitial pneumonia predominantly affecting the cranioventral pulmonary lobes [8,17,18]. Affected animals are susceptible to secondary bacterial infections [19].

The source of the virus is predominantly an infected animal, and the transmission can be directly, via aerosol or close contact, or indirectly, via contaminated surfaces. Eight subgroups of BRSV have been described worldwide and in Brazil, but recently new untyped subgroups were identified [20,21]. There are few studies that have described the distribution of BRSV in Brazil [20,22]. Accordingly additional molecular characterization is needed to understand the circulation of this virus in cattle herds from this country.

Although there are descriptions of BRSV infections in dairy and feedlot cattle [22,23], reports are predominant in calves [24,25], with few outbreaks in adult animals [22]. Additionally, most recent studies that have investigated the occurrence of BRSV in Brazil were based on either serological evaluation [11,12,26], or the molecular aspects of this virus [20,27,28], while descriptions of the clinical and pathological aspects of the associated disease are in older publications [29,30]. Collectively, the data available in Brazil relative to the occurrence of BRSV is scarce when compared to countries where cattle rearing and production is predominant. The objectives of this study were: (1) describe the clinical, pathological, and molecular findings, observed in unvaccinated animals during an outbreak of respiratory disease in a dairy herd from Southern Brazil; (2) provide additional information as to occurrence of BRSV in this continental nation; and (3) investigate the usage of a multiplex qPCR assay as a rapid and reliable method for the detection of respiratory pathogens of cattle. Furthermore, the possible utilization of oral swabs for the detection of agents of BRD was evaluated.

## 2. Materials and Methods

### 2.1. Geographical Location, Clinical Presentation, and Sampling

This outbreak occurred on a dairy farm located within the municipality of Sengés, Southern Brazil, with 17,344 residents and a cattle population of 22,463 [31]. This small municipality is in the Central Eastern Mesoregion of Paraná state, 272 km from Curitiba, the state capital, and it borders the state of São Paulo [31]. The average temperature in Sengés during June and July varies between 11 and 21 °C, with 64–75 mm of rain; however, during this outbreak there was no rain with temperature variation between 6 to 25 °C.

The affected Holstein dairy herd included 48 lactating cows, seven dry cows, 37 heifers, and 13 calves. The lactating cows were housed in a compost barn, heifers were reared under the free stalls management system, and calves were maintained in individual pens. The average milk yield was 31 L/cow per day. Cows at this farm received a mixed ration containing corn silage, commercial feed, and oat grass, which was introduced 15 days before the onset of the respiratory signs; water was provided ad libitum. Additionally, limestone was being pulverized at a neighboring farm two days before the first clinical signs were observed. All cows and heifers were immunized against clostridial diseases, while the female calves were immunized against brucellosis. Cattle at this farm were not immunized against respiratory diseases. The herd was treated with levamisole subcutaneously six months ago. The farmer related that there was no introduction of animals at this farm over the last five years.

The first demonstration of cattle with signs of respiratory disease was observed by the farmer in early June 2023, after which the consulting veterinarian was contacted. Milk yield production decreased drastically. Cows #24 and 25 reportedly had subcutaneous emphysema. The consulting veterinarian treated all affected animals with gentamicin and diclofenac, both intramuscularly, but without any success. Thereafter, the veterinarian contacted the Large Animal Internal Medicine Service of the Veterinary Hospital–Federal University of Paraná to assist with the outbreak.

In mid-June 2023, a team of veterinarians from the Federal University of Paraná visited the farm to investigate the occurrence. During the visit, the team conducted a thorough examination of the herd and gathered information about the outbreak from the farmer. The small size of the herd allowed the visual inspection of all animals, where it was observed that all lactating cows had signs of respiratory disease, such as respiratory distress and cough. The severity of respiratory signs varied across animals, with some displaying more severe signs and others showing less severe manifestations. Samples were not collected from the heifers because this category of animals did not show severe respiratory signs during the clinical evaluation.

The farm did not have a chute or an appropriate place to restrict the animals, which limited the number of cows examined and the samples collected. Accordingly, sampling and clinical examination of all animals were done at the milking room, where only cows with the worst clinical signs during the inspection were selected. Additionally, since all calves had signs of respiratory disease, and were housed individually, samples from all animals within this category were possible.

Clinical examination of sick animals was performed and biological samples were collected for diagnosis. Paired nasal and oral swabs (15 cm length) were collected from three cows and five calves with at least one sign of respiratory disease (nasal discharge, cough, dehydration, and dyspnea). The swab was completely placed into the mouth and moved around for approximately 3 s to obtain as much material as possible. Furthermore, paired nasal and oral swabs were collected from two cows without clinical manifestations of respiratory disease, but with the reported subcutaneous lesions suggestive of emphysema. During the visit at the farm, a 41-month-old Holstein dairy cow (#21), with signs of severe dyspnea, died suddenly. A routine post-mortem evaluation was done immediately after death, during which duplicate fragments of the lungs, trachea, mediastinal lymph nodes, kidneys, myocardium, and liver were collected; one part was maintained refrigerated, and the other fixed by immersion in 10% neutral buffered formalin solution.

All swabs were maintained in a transport medium and refrigerated at 8 °C until used in analyses. The swabs and fresh organ samples were submitted to the Marcos Enrietti Diagnostic Center, Curitiba, Paraná, Brazil for molecular evaluation with a panel of viral and bacterial agents. Organs immersed in 10% buffered formalin solution were sent to the Veterinary Pathology Laboratory, Federal University of Paraná for pathological evaluations. Formalin-fixed paraffin-embedded (FFPE) tissues sections of selected organs were sent to the Laboratory of Animal Pathology, Universidade Estadual de Londrina, for the immunohistochemical (IHC) detection of BRSV antigens.

### 2.2. Histopathological Evaluations and Immunohistochemical Detection of BRSV Antigens

All samples received were routinely processed for histopathological evaluation with the Hematoxylin and Eosin stain. The IHC detection of BRSV antigens was performed on FFPE tissue sections using a previously described protocol [32]. Positive controls consisted of utilizing FFPE tissue sections known to contain antigens of BRSV from previous reports [32,33]. For negative controls, the primary antibody was substituted with its diluent. Negative and positive controls were included in all IHC assays.

### 2.3. Molecular Detection of Infectious Agents Associated with Respiratory Disease of Cattle

Nasal and oral swabs, as well as selected tissue fragments, were submitted to nucleic acid extraction by the Boom method [19]. Oral swabs were collected in duplicate to evaluate their possible use in the identification of agents of respiratory diseases.

A Multiplex real-time (qPCR) assay was designed to detect the nucleic acids of viral and bacterial pathogens associated with the development of BRD. These included: BRSV, BoAHV1, BPIV3, BCoV, OvGHV2, *H. somni*, *P. multocida*, *M. haemolytica*, and *M. bovis*. A list of the specific primers and probes used for the qPCR detection of these pathogens of respiratory disease of cattle is provided in Table 1.

The multiplex qPCR reactions were carried out using 3.7 μL of the nucleic acids by using the commercial kit AgPath-ID One-Step RT-PCR (Thermo Fisher Scientific, Austin, TX, USA), 1.68 µL RNase-free water, 4 µL 2× RT-PCR Buffer, 0.32 µL 25× RT-PCR enzyme mix, 0.3 μM of each primer forward, 0.3 μM of each primer reverse, and 0.3 μM of each hydrolysis probe. A positive control and ultrapure DNase–RNase-free distilled water (Thermo Fisher Scientific, Austin, TX, USA), used as No Template Control, were included in each run. The cycling conditions consisted of 50 °C for 20 min followed by denaturation at 95 °C for 2 min, and 45 cycles of denaturation at 95 °C for 15 s, and annealing, amplification, and detection at 58 °C for 1 min.

All samples with a threshold cycle (*Cq*) of at least 37 were considered positive in their respective qPCR assays. Samples with a *Cq* greater than 37 or without amplification were classified as negative. The qPCR reactions were performed in a 7500 Fast Real-time PCR System (Applied Biosystem, Waltham, MA, USA) in 0.2 mL thin-wall strips or 96-well plates (Thermo Fisher Scientific, Waltham, MA, USA).

Additionally, conventional single-plex PCR assays were performed to amplify the nucleic acids of bovine ephemeral fever virus (BEFV), BVDV, and BRSV. The molecular detection of these pathogens was performed by using the previously described primers for BVDV [38], BEFV [39], and BRSV [40]. BEFV was included in this investigation since this virus was associated with the development of subcutaneous emphysema in ruminants [41]. This specific BRSV RT-PCR assay was performed to obtain larger nucleotide (nt) sequences for phylogenetic analyses to identify the F and G genes.

The conventional PCR assays were performed in a Mastercycler gradient Eppendorf (Eppendorf AG, Hamburg, Germany) with an initial step of 50 °C for 30 min, denaturation step at 95 °C for 10 min followed by 45 cycles of 95 °C for 15 s, 57 °C for 45 s, 72 °C for 30 s and finally 1 cycle of 72 °C for 3 min. Reactions were prepared using 3 μL of RNA with AgPath-ID One-Step RT-PCR (Thermo Fisher Scientific, Austin, TX, USA), 0.8 µL RNase-free water, 5 µL 2× RT-PCR Buffer, 0.4 µL 25× RT-PCR enzyme mix, 0.4 μM of primer forward, and 0.4 μM of primer reverse.

### 2.4. Sanger Sequencing and Phylogenetic Analysis

The amplicons were purified using HT ExoSAP-IT (Thermo Fisher Scientific, Austin, TX, USA) according to the manufacturer’s instructions. The purified amplicons were sequenced in both directions using the BigDye Terminator kit v. 31 (Thermo Fisher Scientific, Austin, TX, USA) and cleaned up with BigDye Xterminator v. 3.1. (Applied Biosystems, Foster City, CA, USA). The purified products of the cycle sequencing were analyzed on the ABI 3130xl Genetic Analyser (Applied Biosystems, Foster City, CA, USA). The phylogenetic analyses were performed by using the Maximum Likelihood method with the partial (422 nt) F gene sequences of BRSV deposited in GenBank. Sequence quality analyses and consensus sequences were obtained using Phred and CAP3 homepages, respectively (http://asparagin.cenargen.embrapa.br/phph/, accessed on 8 October 2025). Similarity searches were performed with nt sequences deposited in the GenBank database using the Basic Local Alignment Search Tool (BLAST) homepage (http://blast.ncbi.nlm.nih.gov/Blast.cgi, accessed on 8 October 2025). Multiple and pairwise alignments with strains available in GenBank were performed with MEGA software version 7.0.26 [42]. The phylogenetic tree was based on nucleotide (nt) sequences using the maximum-likelihood method and Tamura & Nei model [43], which provided statistical support with 1000 bootstrap replicates using the MEGA package (version 7.0) [42]. Sequence identity matrices were performed using the BioEdit software version 7.2.5 [44].

### 2.5. Statistical Analysis

Non-parametric statistical evaluations were performed using the Spearman rank (rho) correlation coefficient to determine possible associations between the nasal and oral shedding of BRSV, *P. multocida*, and *H. somni* during this outbreak. The Paired Wilcoxon test was performed to compare nasal and oral shedding of BRSV. The *p* ≤ 0.05 was considered significant.

## 3. Results

### 3.1. Epidemiological Data and Clinical Findings

**Figure 1:**
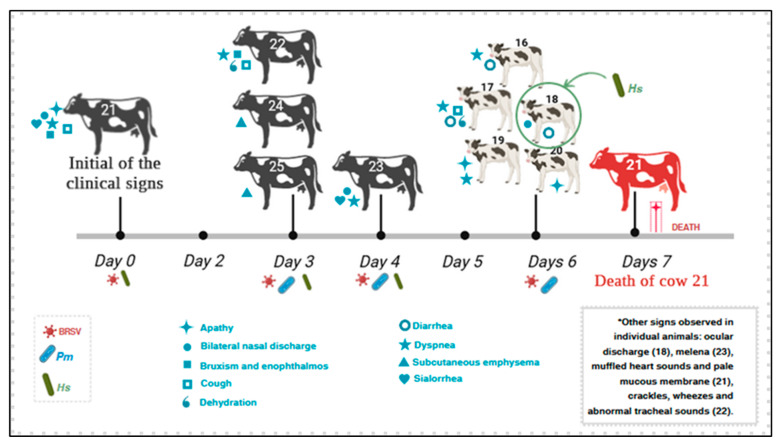
Sequential events observed during an outbreak of respiratory disease affecting adult dairy cows and calves from Southern Brazil. The numbers of the affected animals are provided. *—clinical manifestations reported by the consulting veterinarian. X—cow that died without organs evaluated. Cow # 21 died during the visit to the farm.

The progression of the outbreak is graphically represented in Figure 1. The initial clinical signs reported at the farm were typical of respiratory distress with a marked drop in milk yield of dairy cows. Six days after the onset of pulmonary distress, cutaneous swelling suggestive of emphysema was described in cows #24 and #25. The farmer reported that another cow died with clinical manifestations of respiratory discomfort on this day, but neither biological samples nor tissues were collected for evaluation. The clinical investigation at the farm revealed that all categories of cattle, including calves and adult cows, exhibited at least one clinical sign of respiratory distress, such as coughing, nasal discharge, and labored breathing. Table 2 shows the age and rectal temperature of each animal. Fever was noted in several of these, including cow #25 with cutaneous emphysema. During this outbreak of respiratory disease, morbidity was 100% for calves (13/13) and adult cows (55/55), and 30% (11/35) for heifers. Mortality was low (1.9%; 2/105).

**Figure 2:**
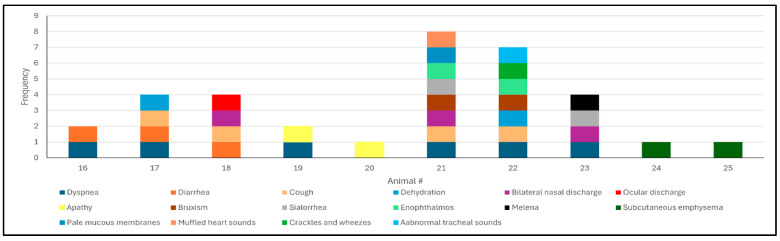
Frequency of clinical manifestations observed in an outbreak of respiratory disease in dairy cattle from Southern Brazil.

**Figure 3:**
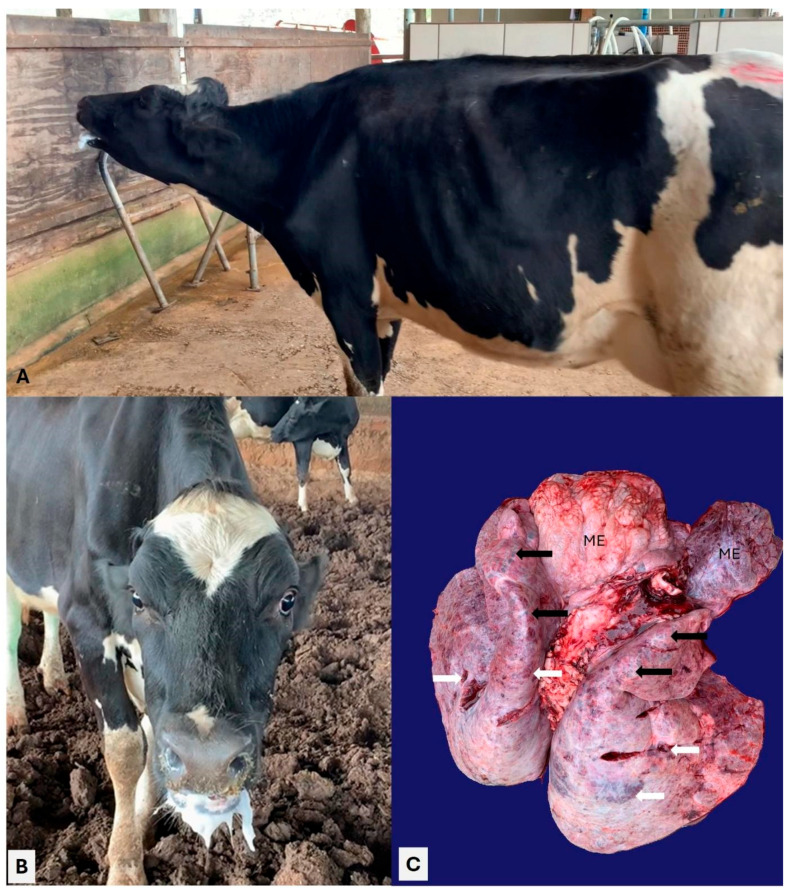
Clinical and gross manifestations of pulmonary disease of cow #21 concomitantly infected by BRSV and *Histophilus somni*. Observe the stretched neck (**A**) with copious, frothy secretion at the oral cavity and evidence of mucopurulent nasal secretion (**B**). There is mediastinal emphysema (ME) adjacent to the lungs (**C**), which has emphysema (black arrows) at the cranioventral lobes and areas of consolidation (white arrows) at the caudal pulmonary lobes.

All clinical manifestations observed in the animals evaluated during this outbreak are summarized in Figure 2. Overall, adult dairy cows were more severely affected relative to dairy calves, with cows #21 and 22 being more severely affected. Dyspnea was the most frequent clinical manifestation observed, being identified in cows #21, 22, and 23 as well as in calves #16, 17, and 19. The cow (#21) that died spontaneously typical clinical signs of severe dyspnea, including a stretched neck, grunting during expiration, open-mouth breathing, and frothy oral secretions (Figure 3A,B); auscultation revealed bilateral crackles and wheezes and muffled heart sounds. Cow #23 with dyspnea was the only animal with melena. Cutaneous swelling that was interpreted as subcutaneous emphysema was reported in cows #24 and 25.

All calves evaluated were prostrated, with clinical manifestations of pulmonary discomfort being more severe in calves #17 and 18, with diarrhea identified in calves #16, 17, and 18. The heifers were less severely affected, with cough being the only clinical sign observed in this category of animals.

### 3.2. Pathological Observations

Two adult cows died during this outbreak of respiratory disease at this farm, one before and the other during the veterinary field investigation. However, the results of the first cow are unknown. Post-mortem evaluation of the second cow (#21) that died revealed severe emphysema and bullae at the cranioventral lobes, with patchy areas of consolidation at the caudoventral pulmonary lobes (Figure 3C). Emphysema was also observed in the mediastinal region next to the lungs.

**Figure 4:**
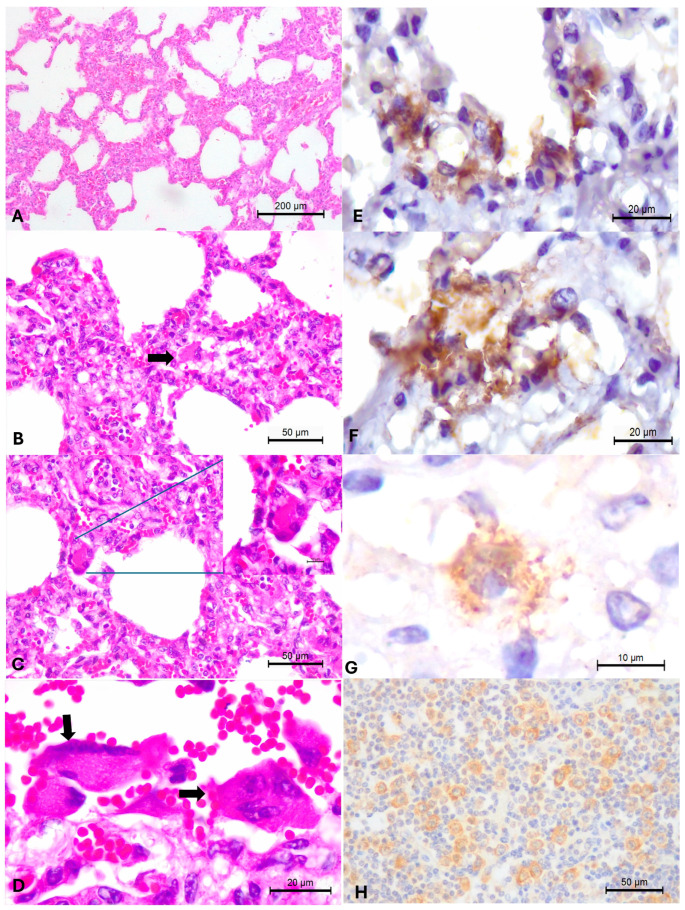
Histopathological and immunohistochemical findings observed in a cow that died of respiratory distress. Observed interstitial pneumonia due to the thickening of alveolar septa without the accumulation of neutrophilic exudate (**A**); a closer view is provided (**B**), containing a multinucleated syncytial (arrow) within the thickened alveolar wall. Intralesional syncytial formation is also demonstrated with a closer provided at the insert (**C**); there are several syncytial cells (arrows) lining the alveolar wall (**D**). There is positive intracytoplasmic immunoreactivity to BRSV antigens within epithelial cells of bronchiole (**E**), and multinucleated giant cells (**F**) of the lungs, as well as within hepatocytes (**G**) and the peribronchial lymph node (**H**). Hematoxylin and eosin stain (**A**–**D**); immunoperoxidase counterstained with Hematoxylin (**E**–**H**). Bars: (**A**), 200 µm; (**B**,**C**,**H**), 50 µm; (**D**–**F**), 20 µm; (**G**), 10 µm.

Histopathology revealed patchy areas of interstitial pneumonia characterized by moderate to severe thickening of the alveolar septa due to the proliferation of type II pneumocytes with a moderate influx of lymphoplasmacytic inflammatory cells, but without the accumulation of neutrophilic exudate within the pulmonary airspaces (Figure 4A,B). Numerous multinucleated syncytial cells were observed next to areas of alveolar thickening (Figure 4C,D). Additional significant pulmonary alterations included severe interstitial emphysema, areas of extensive subpleural hemorrhage, thrombosis, pleuritis, and moderate distension of interlobular septa by exudate. Hyaline formation was not observed in the pulmonary sections evaluated.

### 3.3. Immunohistochemical Identification of BRSV Antigens

The BRSV IHC assay revealed positive, intralesional, intracytoplasmic immunoreactivity within epithelial cells of the lungs of the adult cow that died with histological evidence of interstitial pneumonia with multinucleated syncytial cells. Immunoreactivity occurred within epithelial cells of the pulmonary bronchiole and multinucleated giant cells (Figure 4E,F). There was mild, scanty, positive immunoreactivity to BRSV antigens within hepatocytes of the liver (G) and scattered immunoreactivity within lymphocytes and macrophages of the peribronchiolar lymph node (Figure 4H).

### 3.4. Molecular Detection of Infectious Agents of BRD by Multiplex qPCR

Among the infectious pathogens investigated, only BRSV, *H. somni*, and *P. multocida* were detected in this study. The distribution and corresponding *Cq* values of the three BRD-associated pathogens, detected by multiplex qPCR in the nasal and oral samples from adult cows and dairy calves during this study, are summarized in Table 3.

All nasal samples from both adult cows and dairy calves tested positive for BRSV by qPCR. In contrast, only 70% (7/10) of the oral samples were positive for BRSV, 20% (2/5) in adult cows, and 100% (5/5) in calves. Bacterial infections caused by *H. somni* and *P. multocida* were detected in both adult and young animals, with the highest prevalence observed in adult cows. *H. somni* was detected in 60% (6/10) of the nasal and 50% (5/5) of the oral samples, while *P. multocida* was identified in 70% of the nasal and 90% of the oral samples.

In addition, cow #21 that died with clinical manifestations of respiratory disease, with histological evidence of interstitial pneumonia, and contained BRSV antigens within epithelial cells by IHC had elevated loads of BRSV by qPCR (Table 2) within the nasal (*Cq* 28.42) and oral (*Cq* 35.17) samples, as well as in the lungs (27.03) and trachea (*Cq* 30.24). Furthermore, only identifiable levels of *H. somni* (*Cq* 36.89) close to the cut-off threshold (*Cq* 37) by qPCR were detected in the oral sample from this cow, while the nasal sample contained *H. somni* beyond (*Cq* 37.15) the cutoff threshold. *P. multocida* was not detected in any of the samples evaluated from this cow.

Additionally, the nucleic acids of BoAHV1, BPIV3, BCoV, OvGHV2, *M. haemolytica*, and *M. bovis* were not amplified by multiplex qPCR, and BVDV and BEFV were not detected by conventional singleplex PCR.

### 3.5. Simultaneous Infections Detected in Adult Dairy Cows and Calves with BRD

Concomitant respiratory infections were identified in all animals evaluated during this study, with dual infections occurring predominantly (80%; 4/5) in calves, while triple infections were more frequent (80%; 4/5) in adult dairy cows (Table 3). BRSV occurred in all simultaneous infections: dual infections were associated with BRSV and *P. multocida* (*n* = 4) and BRSV with *H. somni* (*n* = 1). Triple infections due to BRSV, *H. somni*, and *P. multocida* occurred in cows #22, 23, 24, and 25, and in calf #18. As previously indicated, cow #21, which died of BRD, was simultaneously infected by BRSV and *H. somni*. Additionally, when the two bacterial infections were compared (Table 3), cattle were more frequently concomitantly infected by *P. multocida* (90%; 9/10) relative to *H. somni* (60%; 6/10).

The average *Cq* values of the three infectious disease pathogens of BRD identified in the nasal and oral samples during this outbreak are provided in Table 3. The *Cq* values for BRSV within the nasal samples varied from 22.03 (cow#22) to 33.46 (calf #19), with an average *Cq* of 27.43. Moreover, the *Cq* values for BRSV within the oral samples varied between 26.49 (cow# 22) to 36.23 (calf #17). These findings demonstrated that cow#22, which demonstrated the most severe clinical manifestations of pulmonary distress (Table 2), had elevated concentrations of BRSV nucleic acids within the nasal and oral secretions during this investigation.

The average *Cq* values for BRSV and *H. somni* were comparatively lower within the nasal relative to oral samples when all animals evaluated were compared (Table 3). Alternatively, the average *Cq* values for *P. multocida* were more elevated within oral samples as compared to samples obtained from the nasal cavity. These findings demonstrated that the average concentrations of BRSV and *H. somni* were more elevated within the nasal samples, with the load of *P. multocida* being more elevated in oral samples.

### 3.6. Statistical Correlations Between Nasal and Oral Shedding of the Infectious Disease Pathogens Identified in Cattle with Respiratory Disease

**Figure 5:**
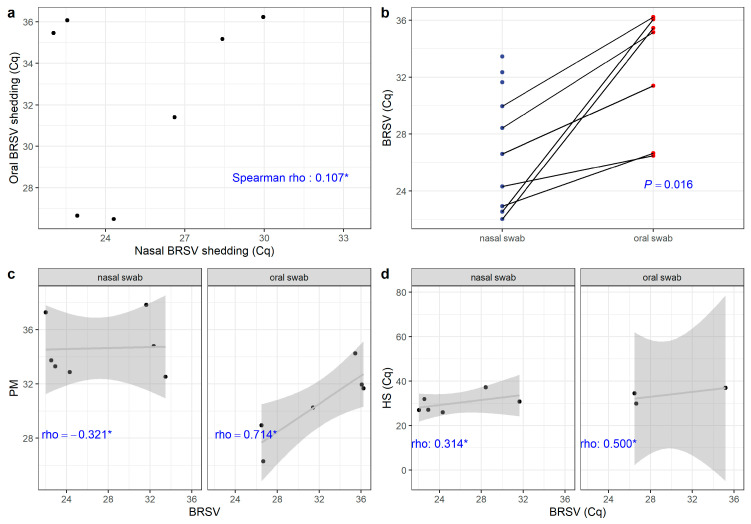
Correlation between nasal and oral shedding of the different respiratory pathogens. (**a**) Spearman correlation between oral and nasal Bovine Respiratory Syncytial Virus (BRSV) shedding (*: not statistically significant). (**b**) Paired samples analysis comparison of nasal and oral BRSV Cq (Paired Wilcoxon test *p*-value). (**c**) Spearman correlation between *Pasteurella multocida* (PM) and BRSV shedding in oral and nasal swabs. (**d**) Spearman correlation between *Histophilus somni* (HS) and BRSV shedding in oral and nasal swabs.

The statistical correlations between the oral and nasal shedding of BRSV, *H. somni*, and *P. multocida* detected during this outbreak of respiratory distress in cattle are graphically demonstrated in Figure 5. A weak correlation (Spearman = 0.107, *p* = 0.84) between the nasal and oral shedding of BRSV (Figure 5a) was observed when the viral loads detected in these animals were compared. However, a significant statistical relationship (*p* = 0.016) was observed when the differences between the nasal and oral secretions of BRSV were compared (Figure 5b).

When the correlation between nasal and oral shedding of the three pathogens identified during this study was evaluated, a negative correlation (Spearman rho, −0.321, *p* = 0.4976) was observed between the nasal shedding of BRSV and *P. multocida* (Figure 5c). Alternatively, there was a positive correlation (Spearman rho, 0.714, *p* = 0.136) between the oral shedding of BRSV and *P. multocida* (Figure 5d).

### 3.7. Molecular Characterization of BRSV and Phylogenetic Analysis of the BRSV Fusion Protein Gene

**Figure 6:**
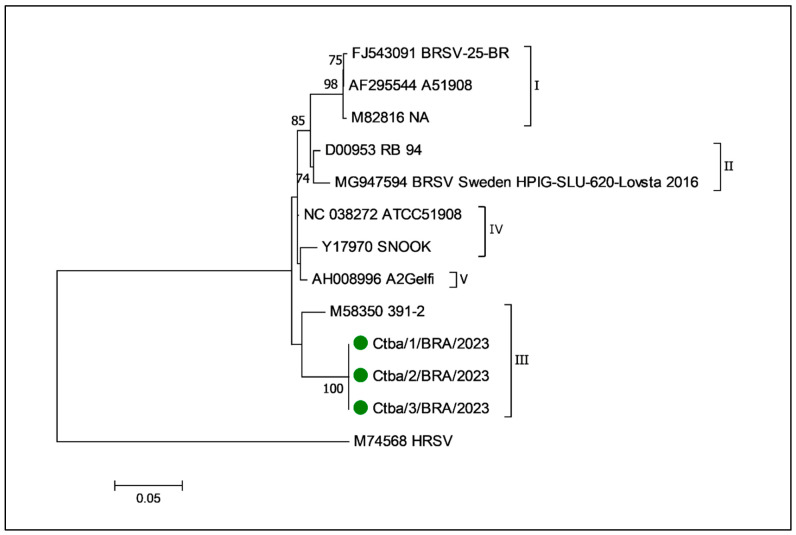
Phylogenetic analysis using the Maximum Likelihood method for the partial (422 nt) F gene sequences of bovine respiratory syncytial virus. The percentage of trees on which the associated taxa clustered together is shown next to the branches. The tree is drawn to scale, with branch lengths measured in the number of substitutions per site. The analysis involved 13 nt sequences from BRSV strains. The human respiratory syncytial virus (HRSV) sequence was used as an outgroup. Evolutionary analyses were performed using MEGA7. The BRSV strains identified in this study are indicated by green circles. The designations at the end of the branches (I–V) refer to the subgroups.

A fragment of 481 bp region encoding F protein and a 371 bp fragment of a region encoding the G protein were amplified by RT-PCR [26]. The targeted amplicons of the BRSV F and G genes were successfully amplified in the RT-PCR assays. Sequencing was only performed with the samples derived from the nasal cavity, lungs, and trachea of cow #21 that died during this outbreak. However, high-quality nt sequences were only obtained with the BRSV F gene, which were included in the phylogenetic analysis. The partial nt sequences of the F gene amplified from cow # 21 were identical and are deposited in GenBank (PV696130, PV696131, and PV696132). Furthermore, the phylogenetic evaluation of the partial fragment of the BRSV F gene (Figure 6) detected in this study revealed that the strain derived from this cow clustered with sequences of the BRSV III subgroup.

## 4. Discussion

The outbreak of respiratory disease in this dairy herd was characterized by 100% morbidity in calves and adult cows, with reduced mortality and 30% morbidity in heifers. Outbreaks of BRSV infections can have morbidity varying between 60-80% [8]. The clinical manifestations observed in cow #21 during this outbreak have been associated with previous descriptions of infections by BRSV [8,29], with disease being more severe in this study since the herd was unvaccinated [45]. However, the additional clinical signs cannot be attributed exclusively to infections by BRSV, since all animals were concomitantly infected by either *H. somni* and/or *P. multocida*. Therefore, the clinical manifestations herein described in the other nine animals were associated with the development of BRD [1,2,3,18]. Nevertheless, the histopathological and IHC findings observed in the cow that died are typical of infections due to BRSV [14,45], demonstrating that the clinical signs of this cow were directly related to pathological evidence of disease. Furthermore, molecular investigations detected BRSV RNA from the nasal and oral samples and in the lung and trachea of cow #21 that died as well from all nasal samples and in 70% (7/10) of the oral samples derived from the other nine animals investigated. Moreover, the concomitant detection of *P. multocida* (90%; 9/10) and/or *H. somni* (60%; 6/10) in the affected animals confirmed that these three pathogens (BRSV, *H. somni*, and *P. multocida*) were associated with the development of the respiratory disease syndrome that occurred in the animals herein described. Accordingly, it is arguable that BRSV was the primary infection during this outbreak, with concomitant secondary bacterial infections due to *H. somni* and *P. multocida*.

Additionally, the non-detection of the nucleic acids of BoAHV1, BPIV3, BVDV, BCoV, OvGHV2, BEFV, *M. haemolytica*, *and M. bovis* from either the nasal or oral samples of all animals investigated suggests that these pathogens were not associated with the development of the clinical syndrome observed in these animals at this farm. Furthermore, BEFV is exotic to Brazil and was only investigated during this study because two cows (#24 and 25) developed subcutaneous emphysema, which is a common gross manifestation of infection due to this agent [41]. Nevertheless, this viral pathogen was not detected in any of the samples evaluated, indicating that BEFV was not associated with the development of the cutaneous manifestations observed in these two cows. However, subcutaneous emphysema, as observed in these cows, has been associated with infections due to BRSV [8,14,18]. Moreover, the elevated viral loads of BRSV detected in the nasal samples derived from cows #24 and #25 (*Ct*, 22.03 and 22.55, respectively) demonstrated a possible relationship between the elevated viral loads and the clinical manifestations observed in these animals.

### 4.1. Adverse Environmental Conditions and Management Practices Were Possible Triggers for This Outbreak

Although the exact reason for this outbreak remains unknown, the summation of existing environmental conditions could have influenced the occurrence of this outbreak. Outbreaks of BRD [3] and BRSV [17] are associated with abrupt changes to environmental conditions and stressors, with outbreaks usually occurring after a drop in temperature, common during the winter [46]. The current outbreak occurred during the winter of the Southern Hemisphere. During this outbreak of respiratory disease, there was a marked increase in the temperature amplitude in Sengés for this period, from a yearly average of 11–21 °C to 6–25 °C without any rain. Therefore, the region was colder than normal and unusually dry. Furthermore, limestone was being pulverized on a neighboring farm two days before the first clinical signs were observed. Additionally, the compost barn management system could have contributed to an increase in dust within the environment due to the high pulverulent bed used in this rearing system. Dust in the air increases during dry climates, and the presence of small particles may cause inflammation to the respiratory tract, carry microorganisms [13], and affect mucociliary clearance [47]. Dust affects cattle health and production and can contribute to the development of respiratory disease [48,49]. Accordingly, these environmental factors collectively could have contributed to the development of the respiratory outbreak herein described.

Another contributory factor that could have been related to this outbreak of the BRD was the absence of immunization against respiratory disease agents at this farm; similar results were previously described [12,50,51,52]. Additionally, unvaccinated cattle infected with BRSV tend to eliminate elevated viral concentrations via nasal secretions [45], as was observed in most cattle during this investigation, where the *Cq* of BRSV varied between 22.03 and 33.46, with an average *Cq* of 27.43.

Interestingly, adult dairy cattle during this outbreak demonstrated clinical manifestations of respiratory distress approximately two weeks after receiving feed containing fresh oat grass; similar disease progression was described in atypical or acute interstitial pneumonia (AIP) of feedlot cattle [48,53,54]. AIP may occur after beef cattle have ingested a wide range of green pastures [54], with clinical manifestations of disease occurring within 2–3 weeks after the alteration of diet composition [53]. Morbidity due to AIP can affect more than 50% of the affected herd, while mortality varies between 30 and 100% [54], occurring 21–73 days after the onset of respiratory disease [53]. However, in the current investigation, morbidity was elevated, but mortality was extremely reduced. Additionally, in this case, there was the absence of key histological elements to effectively diagnose AIP, particularly hyaline membrane formation and proteinaceous-rich fluid within alveoli [54,55,56]. Furthermore, there seems to be an overlap between the histopathological diagnostic features of BRSV-associated interstitial pneumonia and AIP [56]. Accordingly, in this investigation, BRSV-induced interstitial pneumonia was diagnosed based on the molecular and immunohistochemical detection of viral nucleic acids and antigens, respectively, in cow #21, and the non-detection of other viral pathogens associated with the development of BRD in the other animals evaluated. Nevertheless, BRSV is incriminated as one of the known causes associated with AIP [48,54].

### 4.2. Simultaneous Infections Are Frequent in Outbreaks of Bovine Respiratory Disease

The qPCR assay used during this study was efficient for the detection of pathogens associated with BRD and can serve as a routine diagnostic assay to evaluate and quantify the occurrence of respiratory disease pathogens in cattle from Brazil. During this investigation, all animals were concomitantly infected by at least two pathogens associated with the development of BRD, with infections by BRSV being detected in all animals. Concomitant infections by a wide range of bacterial and viral agents of respiratory disease have been described in cattle from Brazil [3,5,12,27,28,32,33] and other countries [13,50,52,57]. The constant demonstration of multiple disease pathogens in cattle with BRD worldwide demonstrates the multietiological nature of this disease entity and the complexity associated with the diagnosis of respiratory infections in cattle.

The detection of multiple pathogens in dairy cattle during this study using qPCR is a turning point for the diagnosis of respiratory infection of cattle in Brazil, since this study represents the first investigation to effectively evaluate the viral loads of cattle with BRD from this continental nation by a multiplex qPCR system. All previous studies performed in Brazil used either conventional simple-plex molecular diagnostic or serological assays to assess the occurrence of infectious respiratory disease pathogens of cattle. Consequently, this multiplex qPCR diagnostic assay will drastically reduce the related costs and turnaround time associated with the identification of BRD pathogens in Brazil, which are of fundamental importance for the assessment and understanding of infectious disease dynamics in this country.

During this investigation, cow #21, which died with clinical manifestations of severe dyspnea, had histological evidence of interstitial pneumonia, contained BRSV antigens by IHC, had an evaluated viral load of BRSV by qPCR, and was simultaneously infected by *H. somni*. However, the bacterial load of *H. somni* detected in the oral cavity of this animal was close to the cut-off threshold, suggesting that the bacterial concentration was very low and may not have been sufficient to induce pulmonary disease. This can be correlated with the absence of typical patterns of bacterial-induced bronchopneumonia in the pulmonary sections of the cow evaluated.

### 4.3. BRSV Shedding in Nasal and Oral Samples

During this study the pathogens identified were detected in the nasal and oral samples, with no statistical difference being associated with their occurrence in either anatomical location. Although the average *Cq* value of BRSV detected in nasal samples (27.43) was comparatively lower than that observed in oral samples (32.68), suggesting a higher viral load in the nasal as compared to oral samples, no statistical difference was detected when these values were compared. However, a significant statistical association (*p* = 0.016) was observed when the differences between the nasal and oral secretions of BRSV were compared. This result may suggest that either route can be efficiently used for the detection of BRSV in cattle with BRD by qPCR assays. However, caution must be taken with the interpretation of this result since the number of animals evaluated during this study was relatively reduced. Consequently, a larger number of animals must be evaluated to demonstrate the effectiveness of this relationship.

Nevertheless, these initial findings demonstrated that BRSV, and to some extent *H. somni* and *P. multocida*, can be detected in the oral cavity of cattle with clinical manifestations of respiratory disease, suggesting that this route may serve as an alternative for the collection of field samples in cattle with respiratory distress, principally in animals with copious nasal secretions. Accordingly, an analysis of a larger population of paired nasal and oral samples of cattle with BRD should be performed to attest to the real suitability of using oral samples for the diagnosis of respiratory diseases in cattle. However, in human medicine, the utilization of paired nasal and throat [58] or oropharyngeal [59] swabs seems to detect a larger number of viral agents in persons with respiratory diseases.

Interestingly, low *Cq* values of these three agents (BRSV, 26.49; *H. somni*, 28.89; and *P. multocida*, 26.3) were detected in the oral samples during this investigation, indicating the possibility of using oral samples for the diagnosis of agents of BRD; therefore, these results suggest that both oral and nasal swabs can be used for the molecular detection of these agents in cattle with respiratory disease.

### 4.4. Phylogenetic Relationships of the BRSV Fusion Protein Gene

The phylogenetic analysis revealed that the BRSV wild-type strains identified in cattle during this study clustered with strains from subgroup III. Additionally, a phylogenetic study that evaluated the wildtype strains of BRSV identified in cattle from the northwest region of Paraná state revealed that the strains formed a distinct unclassified clade [20]. Collectively, these findings may suggest that different wildtype strains of BRSV may be circulating within the same state of Southern Brazil. However, the region of the F gene targeted during this study (113–593) was somewhat distinct from that of the previous investigation (433–1291) [20], with an overlapping of only 113 nt. These differences could have also contributed to the identification of relatively few samples of the same region of the BRSV F gene deposited in GenBank. Accordingly, the results of these two studies cannot be effectively compared. Furthermore, the phylogenetic analysis was based only on the BRSV F gene, since quality sequences were not obtained with the G gene, despite several frustrating attempts; similar findings were described [21].

Consequently, since good quality nt sequences were only obtained for the BRSV F protein gene and the consequent phylogenetic relationship was restricted, we are sequencing the whole genome derived from this study to obtain quality sequences for a more robust phylogenetic analysis. Whole genome sequencing of a strain of BRSV from Brazil will reveal information relative to the pathogenesis, infectability, and variability of this strain and provide accurate comparisons with the genomes of BRSV from other geographical locations to identify possible differences between strains.

### 4.5. Study Limitations

There were two main setbacks during the realization of this study. The collection of a larger number of nasal and oral samples from cattle during this outbreak, including asymptomatic animals and heifers, would have provided a better understanding of the relationship and the dynamics of the infections between the detection of BRSV from the nasal and oral cavity of cattle during outbreaks of BRD and possible associations with the concomitant shedding of *H. somni* and *P. multocida*. Nevertheless, these findings seem to indicate that the oral cavity may be an alternative to obtain samples for the molecular detection of BRSV, and possibly *H. somni* and *P. multocida*, considering the difficulties that may occur during the acquisition of nasal samples from cattle with severe accumulations of nasal secretion. The other setback was the targeting of a region of the BRSV F gene that is not frequently used in the molecular detection of this agent. This prevented the evaluation of a region of the gene that is commonly used in diagnostics and phylogenetic analysis. Nevertheless, whole genome sequencing of the BRSV strain detected during this investigation will provide valuable information about this pathogen in Brazil.

## 5. Conclusions

A multiplex qPCR assay detected BRSV from nasal and oral samples derived from adult dairy cows and calves during an outbreak of respiratory disease. Additionally, there were dual infections due to BRSV with *H. somni* and *P. multocida*, and triple infections associated with these three agents. Furthermore, one adult cow that died after presenting severe respiratory distress had interstitial pneumonia with syncytial cells and contained intralesional BRSV antigens by immunohistochemistry within the lungs and other tissues. The multiplex qPCR assay efficiently quantified the viral and bacterial loads detected in the nasal and oral samples of cattle with clinical manifestations of BRD and in the lung and trachea of the cow that died. This diagnostic method is fast and effective for the identification of respiratory agents of cattle and will contribute to the understanding of these pathogens in Brazil.

## Figures and Tables

**Table 1:** Primers and probes sequences employed in a multiplex real-time PCR (qPCR) system for the detection of viral and bacterial agents of pulmonary disease of cattle.

Agent	Primer/ Probe	DNA Sequence (5′-3′) Probe Labels	References
	F	GGTCAAACTAAATGACACTTTCAACAAG	
BRSV ^1^	R	AGCATACCACACAACTTATTGAGATG	[34]
	P	TGATACAGGTGACAA	
	F	TGTGGACCTAAACCTCACGGT	
BoAHV1 ^2^	R	GTAGTCGAGCAGACCCGTGTC	[34]
	P	AGGACCGCGAGTTCTTGCCGC	
	F	TGATTGGATGTTCGGGAGTGA	
BPIV3 ^3^	R	AGAATCCTTTCCTCAATCCTGATATACT	[34]
	P	TACAATCGAGGATCTTGTTCA	
	F	CTGGAAGTTGGTGGAGTT	
BCoV ^4^	R	ATTATCGGCCTAACATACATC	[35]
	P	CCTTCATATCTATACACATCAAGTTGTT	
	F	CACACCCAACTGGAGTATGAC	
OvGHV2 ^5^	R	ATGTTGTAGTGGGGCCAGTC	[36]
	P	ATGTGCGCTTCGACCCTC	
*Histophilus somni*	F	AAGGCCTTCGGGTTGTAAAG	
	R	CCGGTGCTTCTTCTGTGATTAT	[37]
	P	CGGTGATGAGGAAGGCGATTAG	
*Pasteurella multocida*	F	GGGCTTGTCGGTAGTCTTT	
	R	CGGCAAATAACAATAAGCTGAGTA	[37]
	P	CGGCGCAACTGATTGGACGTTATT	
*Mannheimia haemolytica*	F	ATTAGTGGGTTGTCCTGGTTAG	
	R	GCGTGATTTCGGTTCAGTTG	[37]
	P	CTGAACCAACACGAGTAGTCGCTGC	
*Mycoplasma bovis*	F	TCAAGGAACCCCACCAGAT	
	R	AGGCAAAGTCATTTCTAGGTGCAA	[37]
	P	TGGCAAACTTACCTATCGGTGACCCT	

^1^ BRSV: Bovine Respiratory Syncytial Virus; ^2^ BoAHV1: Bovine alphaherpesvirus 1; ^3^ BPIV3: Bovine Parainfluenza 3; ^4^ BCoV: Bovine coronavirus; ^5^ OvGHV2: Ovine gammaherpesvirus 2; F: primer forward, R: primer reverse, P: probe.

**Table 2:** Biological data and quantification cycle (*Cq*) values for qPCR of endogenous internal control and infectious agents observed in dairy cattle with respiratory disease.

Animal	Age (Months)	Rectal Temperature	Biological Sample	qPCR Threshold Cycle (*Cq*) by Sample			
				β-Actin ^1^	BRSV ^2^	HS ^3^	PM ^4^
16	3	38.6 °C	nasal swab	21.74	26.61	ND	ND
			oral swab	32.42	31.4	ND	30.26
17	2	37.9 °C	nasal swab	22.92	29.96	ND	ND
			oral swab	29.67	36.23	ND	31.67
18	2.5	38.8 °C	nasal swab	23.09	31.64	30.72	37.83
			oral swab	26.18	ND	28.89	31.24
19	5.5	38.4 °C	nasal swab	20.84	33.46	ND	32.53
			oral swab	36.33	ND	ND	35.77
20	2.6	38.8 °C	nasal swab	16.29	32.34	ND	34.79
			oral swab	32.79	ND	ND	27.99
21 ^a^	41	37.7 °C	nasal swab	18.49	28.42	37.15	ND
			oral swab	33.21	35.17	36.89	ND
			lung	25.07	27.03	ND	ND
			trachea	24.99	30.24	ND	ND
			heart, kidney, liver	25	ND	ND	ND
22	50	37.2 °C	nasal swab	21.04	24.31	25.97	32.88
			oral swab	31.07	26.49	34.41	28.95
23	48	37.8 °C	nasal swab	20.02	22.93	27.07	33.31
			oral swab	27.69	26.66	29.85	26.3
24	52	37.1 °C	nasal swab	18.97	22.03	27	37.29
			oral swab	30.77	35.46	ND	34.25
25	49	41 °C	nasal swab	22.26	22.55	31.88	33.75
			oral swab	32.90	36.07	ND	31.95

^1^ β-Actin: endogenous internal control; ND: Not detected; ^2^ BRSV: Bovine Respiratory Syncytial Virus; ^3^ HS: *Histophilus somni*; ^4^ PM: *Pasteurella multocida*. ^a^: adult cow that died during this outbreak.

**Table 3:** Detection of concomitant infections with variations in the threshold cycle (*Cq*) values by qPCR from nasal and oral samples of dairy cattle with respiratory disease.

Animal	Type of Sample				Type of Infection
	Nasal		Oral		
16	BRSV		BRSV + PM		BRSV + PM
17	BRSV		BRSV + PM		BRSV + PM
18	BRSV + HS		HS + PM		BRSV + HS + PM
19	BRSV + PM		PM		BRSV + PM
20	BRSV + PM		PM		BRSV + PM
21	BRSV		BRSV + HS		BRSV + HS
22	BRSV + HS + PM		BRSV + HS + PM		BRSV + HS + PM
23	BRSV + HS + PM		BRSV + HS + PM		BRSV + HS + PM
24	BRSV + HS		BRSV + PM		BRSV + HS + PM
25	BRSV + HS + PM		BRSV + PM		BRSV + HS + PM
		Nasal *Cq* values (average)		Oral *Cq* values (average)	
BRSV		22.03–33.46 (27.43)		26.49–36.23 (32.68)	
*Histophilus somni*		25.97–31.88 (29.96)		28.89–36.89 (32.51)	
*Pasteurella multocida*		32.53–34.79 (34.62)		26.3–35.77 (30.93)	

Legend: BRSV: Bovine Respiratory Syncytial Virus; HS: *Histophilus somni*; PM: *Pasteurella multocida*.

## Data Availability

The nucleotide sequence of the BRSV strains identified during this study is deposited in GenBank (https://www.ncbi.nlm.nih.gov/genbank/, accessed on 9 October 2025).

## References

[1] Fulton R.W. (2009). Bovine Respiratory Disease Research (1983–2009). Anim. Health Res. Rev..

[2] Cooper V.L., Brodersen B.W. (2010). Respiratory Disease Diagnostics of Cattle. Vet. Clin. N. Am. Food. Anim. Pract..

[3] de Castro M.M., de Oliveira T.E.S., Headley S.A. (2021). Bovine Respiratory Disease in Brasil: A Short Review. Semin-Cienc. Agrar..

[4] Smith R.A., Step D.L., Woolums A.R. (2020). Bovine Respiratory Disease: Looking Back and Looking Forward, What Do We See?. Vet. Clin. N. Am. Food. Anim. Pract..

[5] Headley S.A., Dall Agnol A.M., Bessegato J.A., Frucchi A.P.S., Maturana É.F.L., Rodrigues R.V., Xavier A.A.C., Alfieri A.F., Alfieri A.A. (2023). Association of Ovine Gammaherpesvirus 2 with an Outbreak of Acute Respiratory Disease in Dairy Cattle. Sci. Rep..

[6] (2025). International Committee on Taxonomy of Viruses (ICTV). https://ictv.global/taxonomy/.

[7] Sarmiento-Silva R.E., Nakamura-Lopez Y., Vaughan G. (2012). Epidemiology, Molecular Epidemiology and Evolution of Bovine Respiratory Syncytial Virus. Viruses.

[8] Valarcher J.F., Ttaylor G. (2007). Bovine Respiratory Syncytial Virus Infection. Vet. Res..

[9] Gonçalves I.P.D., Simanke A.T., Jost H.C., Hötzel I., Dal Soglio A., Moojen V. (1993). Detection of Bovine Respiratory Syncytial Virus in Calves of Rio Grande Do Sul, Brazil. Cienc. Rural.

[10] Campalans J.B., Arns C.W. (1997). Serological Evidence of Bovine Respiratory Syncytial Virus in Brazil. Virus Rev. Res..

[11] Hoppe I.B.A.L., de Medeiros A.S.R., Arns C.W., Samara S.I. (2018). Bovine Respiratory Syncytial Virus Seroprevalence and Risk Factors in Non-Vaccinated Dairy Cattle Herds in Brazil. BMC Vet. Res..

[12] Yoshitani G.D., Camilo S.L.O., Fritzen J.T.T., Oliveira M.V., Lorenzetti E., Lisbôa J.A.N., Alfieri A.F., Alfieri A.A. (2024). Serological Profile for Major Respiratory Viruses in Unvaccinated Cows from High-Yielding Dairy Herds. Animals.

[13] Padalino B., Cirone F., Zappaterra M., Tullio D., Ficco G., Giustino A., Ndiana L.A., Pratelli A. (2021). Factors Affecting the Development of Bovine Respiratory Disease: A Cross-Sectional Study in Beef Steers Shipped From France to Italy. Front. Vet. Sci..

[14] Sacco R.E., McGill J.L., Pillatzki A.E., Palmer M.V., Ackermann M.R. (2014). Respiratory Syncytial Virus Infection in Cattle. Vet. Pathol..

[15] İnce Ö.B., Şevik M., Özgür E.G., Sait A. (2022). Risk Factors and Genetic Characterization of Bovine Respiratory Syncytial Virus in the Inner Aegean Region, Turkey. Trop. Anim. Health Prod..

[16] Bidokhti M.R.M., Tråvén M., Fall N., Emanuelson U., Alenius S. (2009). Reduced Likelihood of Bovine Coronavirus and Bovine Respiratory Syncytial Virus Infection on Organic Compared to Conventional Dairy Farms. Vet. J..

[17] Larsen L.E. (2000). Bovine Respiratory Syncytial Virus (BRSV): A Review. Acta Vet. Scand..

[18] Spilki F.R., Arns C.W. (2008). Vírus Respiratório Sincicial Bovino. Acta Sci. Vet..

[19] Zhou Y., Shao Z., Dai G., Li X., Xiang Y., Jiang S., Zhang Z., Ren Y., Zhu Z., Zhang G. (2023). Pathogenic Infection Characteristics and Risk Factors for Bovine Respiratory Disease Complex Based on the Detection of Lung Pathogens in Dead Cattle in Northeast China. J. Dairy Sci..

[20] Leme R.A., Dall Agnol A.M., Balbo L.C., Pereira F.L., Possatti F., Alfieri A.F., Alfieri A.A. (2020). Molecular Characterization of Brazilian Wild-Type Strains of Bovine Respiratory Syncytial Virus Reveals Genetic Diversity and a Putative New Subgroup of the Virus. Vet. Q..

[21] Affonso I.B., De Souza A., Martini M.C., Dos Santos M.M.A.B., Spilki F.R., Arns C.W., Samara S.I. (2014). Detection of an Untyped Strain of Bovine Respiratory Syncytial Virus in a Dairy Herd. Semin-Cienc. Agrar..

[22] Giammarioli M., Mangili P., Nanni A., Pierini I., Petrini S., Pirani S., Gobbi P., De Mia G.M. (2020). Highly Pathogenic Bovine Respiratory Syncytial Virus Variant in a Dairy Herd in Italy. Vet. Med. Sci..

[23] Ferella A., Streitenberger N., Pérez Aguirreburualde M.S., Dus Santos M.J., Fazzio L.E., Quiroga M.A., Zanuzzi C.N., Asin J., Carvallo F., Mozgovoj M.V. (2023). Bovine Respiratory Syncytial Virus Infection in Feedlot Cattle Cases in Argentina. J. Vet. Diagn. Investig..

[24] Peixoto P.V., Mota R.A., Brito M.F., Corbellini L.G., Driemeier D., Souza M.I.d. (2000). Infecção Natural Pelo Vírus Sincicial Respiratório Bovino (BRSV) No Estado de Alagoas. Pesqui. Vet. Bras..

[25] Larsen L.E., Tegtmeier C., Pedersen E. (2001). Bovine Respiratory Syncytial Virus (BRSV) Pneumonia in Beef Calf Herds Despite Vaccination. Acta Vet. Scand..

[26] Arns C.W., Campalans J., Costa S.C.B., Domingues H.G., D’Arce R.C.F., Almeida R.S. (2003). Characterization of Bovine Respiratory Syncytial Virus Isolated in Brazil. Braz. J. Med. Biol. Res..

[27] Headley S.A., Okano W., Balbo L.C., Marcasso R.A., Oliveira T.E., Alfieri A.F., Negri Filho L.C., Michelazzo M.Z., Rodrigues S.C., Baptista A.L. (2018). Molecular Survey of Infectious Agents Associated with Bovine Respiratory Disease in a Beef Cattle Feedlot in Southern Brazil. J. Vet. Diagn. Investig..

[28] Fritzen J.T.T., Yasumitsu C.Y., Silva I.V., Lorenzetti E., Alfieri A.F., Alfieri A.A. (2024). Respiratory Illness in Young and Adult Cattle Caused by Bovine Viral Diarrhea Virus Subgenotype 2b in Singular and Mixed Bacterial Infection in a BVDV-Vaccinated Dairy Herd. Braz. J. Microbiol..

[29] Driemeier D., Gomes M.J.P., Moojen V., Arns C.W., Vogg G., Kessler L., Costa U.M. (1997). da Manifestação Clínico-Patológica de Infecção Natural Pelo Vírus Respiratório Sincicial Bovino (BRSV) Em Bovinos de Criação Extensiva No Rio Grande Do Sul, Brasil. Pesqui. Vet. Bras..

[30] Flores E.F., Weiblen R., Medeiros M., Botton S.A., Irigoyen L.F., Driemeier D., Schuch L.F., Moraes E.M. (2000). A Retrospective Search for Bovine Respiratory Syncytial Virus (BRSV) Antigens in Histological Specimens by Immunofluorescence and Immunohistochemistry. Pesqui. Vet. Bras..

[31] (2025). Instituto Brasileiro de Geografia e Estatística (IBGE). https://www.ibge.gov.br/cidades-e-estados/pr/senges.html.

[32] Oliveira V.H., Dall Agnol A., Fritzen J.T., Lorenzetti E., Alfieri A., Alfieri A. (2020). Microbial Diversity Involved in the Etiology of a Bovine Respiratory Disease Outbreak in a Dairy Calf Rearing Unit. Comp. Immunol. Microbiol. Infect. Dis..

[33] Oliveira T.E.S., Scuisato G.S., Pelaquim I.F., Cunha C.W., Cunha L.S., Flores E.F., Pretto-Giordano L.G., Lisbôa J.A.N., Alfieri A.A., Saut J.P.E. (2021). The Participation of a Malignant Catarrhal Fever Virus and Mycoplasma Bovis in the Development of Single and Mixed Infections in Beef and Dairy Cattle With Bovine Respiratory Disease. Front. Vet. Sci..

[34] Thonur L., Maley M., Gilray J., Crook T., Laming E., Turnbull D., Nath M., Willoughby K. (2012). One-Step Multiplex Real Time RT-PCR for the Detection of Bovine Respiratory Syncytial Virus, Bovine Herpesvirus 1 and Bovine Parainfluenza Virus 3. BMC Vet. Res..

[35] Decaro N., Elia G., Campolo M., Desario C., Mari V., Radogna A., Colaianni M.L., Cirone F., Tempesta M., Buonavoglia C. (2008). Detection of Bovine Coronavirus Using a TaqMan-Based Real-Time RT-PCR Assay. J. Virol. Methods.

[36] Cunha C.W., Otto L., Taus N.S., Knowles D.P., Li H. (2009). Development of a Multiplex Real-Time PCR for Detection and Differentiation of Malignant Catarrhal Fever Viruses in Clinical Samples. J. Clin. Microbiol..

[37] Kishimoto M., Tsuchiaka S., Rahpaya S.S., Hasebe A., Otsu K., Sugimura S., Kobayashi S., Komatsu N., Nagai M., Omatsu T. (2017). Development of a One-Run Real-Time PCR Detection System for Pathogens Associated with Bovine Respiratory Disease Complex. J. Vet. Med. Sci..

[38] Weinstock D., Bhudevi B., Castro A.E. (2001). Single-Tube Single-Enzyme Reverse Transcriptase PCR Assay for Detection of Bovine Viral Diarrhea Virus in Pooled Bovine Serum. J. Clin. Microbiol..

[39] Gao S., Du J., Tian Z., Niu Q., Huang D., Wang J., Luo J., Liu G., Yin H. (2020). A SYBR Green I–Based Quantitative RT-PCR Assay for Bovine Ephemeral Fever Virus and Its Utility for Evaluating Viral Kinetics in Cattle. J. Vet. Diagn. Investig..

[40] Vilcek S., Elvander M., Ballagi-Pordany A., Belak S. (1994). Development of Nested PCR Assays for Detection of Bovine Respiratory Syncytial Virus in Clinical Samples. J. Clin. Microbiol..

[41] Theodoridis A., Coetzer J.A. (1979). Subcutaneous and Pulmonary Emphysema as Complications of Bovine Ephemeral Fever. Onderstepoort J. Vet. Res..

[42] Kumar S., Stecher G., Tamura K. (2016). MEGA7: Molecular Evolutionary Genetics Analysis Version 7.0 for Bigger Datasets. Mol. Biol. Evol..

[43] Tamura K., Nei M. (1993). Estimation of the Number of Nucleotide Substitutions in the Control Region of Mitochondrial DNA in Humans and Chimpanzees. Mol. Biol. Evol..

[44] Hall T.A. (1999). BioEdit: A User-Friendly Biological Sequence Alignment Editor and Analysis Program for Windows 95/98/NT. Nucleic Acids Symp. Ser..

[45] Makoschey B., Berge A.C. (2021). Review on Bovine Respiratory Syncytial Virus and Bovine Parainfluenza—Usual Suspects in Bovine Respiratory Disease—A Narrative Review. BMC Vet. Res..

[46] Pirie H.M., Petrie L., Pringle C.R., Allen E.M., Kennedy G.J. (1981). Acute Fatal Pneumonia in Calves Due to Respiratory Syncytial Virus. Vet. Rec..

[47] Caswell J.L., Hewson J., Slavić D., DeLay J., Bateman K. (2012). Laboratory and Postmortem Diagnosis of Bovine Respiratory Disease. Vet. Clin. N. Am. Food. Anim. Pract..

[48] Woolums A.R. (2015). Feedlot Acute Interstitial Pneumonia. Vet. Clin. N. Am. Food. Anim. Pract..

[49] Urso P.M., Turgeon A., Ribeiro F.R.B., Smith Z.K., Johnson B.J. (2021). Review: The Effects of Dust on Feedlot Health and Production of Beef Cattle. J. Appl. Anim. Res..

[50] Saa L.R., Perea A., Jara D.V., Arenas A.J., Garcia-Bocanegra I., Borge C., Carbonero A. (2012). Prevalence of and Risk Factors for Bovine Respiratory Syncytial Virus (BRSV) Infection in Non-Vaccinated Dairy and Dual-Purpose Cattle Herds in Ecuador. Trop. Anim. Health Prod..

[51] Chicoski L.M., Fritzen J.T.T., Lorenzetti E., da Costa A.R., Moro E., de Carvalho E.R., Alfieri A.F., Alfieri A.A. (2023). Serological Profile of Respiratory Viruses in Unvaccinated Steers upon Their Arrival at Brazilian Feedlot Facilities. Braz. J. Microbiol..

[52] Lachowicz-Wolak A., Klimowicz-Bodys M.D., Płoneczka-Janeczko K., Bednarski M., Dyba K., Knap P., Rypuła K. (2024). Simultaneous Presence of Antibodies against Five Respiratory Pathogens in Unvaccinated Dairy Calves from South-Western Poland. Animals.

[53] Panciera R.J., Confer A.W. (2010). Pathogenesis and Pathology of Bovine Pneumonia. Vet. Clin. N. Am. Food. Anim. Pract..

[54] Haydock L.A.J., Fenton R.K., Sergejewich L., Squires E.J., Caswell J.L. (2022). Acute Interstitial Pneumonia and the Biology of 3-Methylindole in Feedlot Cattle. Anim. Health Res. Rev..

[55] Sorden S.D., Kerr R.W., Janzen E.D. (2000). Interstitial Pneumonia in Feedlot Cattle: Concurrent Lesions and Lack of Immunohistochemical Evidence for Bovine Respiratory Syncytial Virus Infection. J. Vet. Diagn. Investig..

[56] Chien R.C., Sorensen N.J., Payton M.E., Confer A.W. (2022). Comparative Histopathology of Bovine Acute Interstitial Pneumonia and Bovine Respiratory Syncytial Virus-Associated Interstitial Pneumonia. J. Comp. Pathol..

[57] Gagea M.I., Bateman K.G., Van Dreumel T., McEwen B.J., Carman S., Archambault M., Shanahan R.A., Caswell J.L. (2006). Diseases and Pathogens Associated with Mortality in Ontario Beef Feedlots. J. Vet. Diagn. Investig..

[58] Benkouiten S., Gautret P., Belhouchat K., Drali T., Nougairede A., Salez N., Memish Z.A., Al Masri M., Raoult D., Brouqui P. (2015). Comparison of Nasal Swabs with Throat Swabs for the Detection of Respiratory Viruses by Real-Time Reverse Transcriptase PCR in Adult Hajj Pilgrims. J. Infect..

[59] Kim C., Ahmed J.A., Eidex R.B., Nyoka R., Waiboci L.W., Erdman D., Tepo A., Mahamud A.S., Kabura W., Nguhi M. (2011). Comparison of Nasopharyngeal and Oropharyngeal Swabs for the Diagnosis of Eight Respiratory Viruses by Real-Time Reverse Transcription-PCR Assays. PLoS ONE.

